# COVI-Prim international: Similarities and discrepancies in the way general practices from seven different countries coped with the COVID-19 pandemic

**DOI:** 10.3389/fpubh.2022.1072515

**Published:** 2022-12-06

**Authors:** Andrea Siebenhofer, Anna Mae Scott, Alexander Avian, András Terebessy, Karola Mergenthal, Dagmar Schaffler-Schaden, Herbert Bachler, Sebastian Huter, Erika Zelko, Amanda Murray, Michelle Guppy, Giuliano Piccoliori, Sven Streit, Klaus Jeitler, Maria Flamm

**Affiliations:** ^1^Institute of General Practice and Evidence-Based Health Services Research, Medical University of Graz, Graz, Austria; ^2^Institute of General Practice, Johann Wolfgang Goethe University Frankfurt, Frankfurt, Germany; ^3^Institute for Evidence-Based Healthcare, Bond University, Gold Coast, QL, Australia; ^4^Institute for Medical Informatics, Statistics and Documentation, Medical University of Graz, Graz, Austria; ^5^Department of Public Health-Faculty of Medicine, Semmelweis University, Budapest, Hungary; ^6^Institute for General Practice, Family Medicine and Preventive Medicine, Paracelsus Medical University, Salzburg, Austria; ^7^Institute of General Practice, Medical University of Innsbruck, Innsbruck, Austria; ^8^Faculty of Medicine, Johannes Kepler University of Linz, Linz, Austria; ^9^School of Rural Medicine and New England GP Research Network, University of New England, Armidale, NSW, Australia; ^10^Institute for Special Training in General Medicine, Institute of General Practice, Claudiana Bozen, Bolzano, Italy; ^11^Institute of Primary Health Care (BIHAM), University of Bern, Bern, Switzerland

**Keywords:** COVID-19, general practitioner (GP), public health, self-confidence, perception of risk

## Abstract

**Objectives:**

General practitioners (GPs) are frequently patients' first point of contact with the healthcare system and play an important role in identifying, managing and monitoring cases. This study investigated the experiences of GPs from seven different countries in the early phases of the COVID-19 pandemic.

**Design:**

International cross-sectional online survey.

**Setting:**

General practitioners from Australia, Austria, Germany, Hungary, Italy, Slovenia and Switzerland.

**Participants:**

Overall, 1,642 GPs completed the survey.

**Main outcome measures:**

We focused on how well-prepared GPs were, their self-confidence and concerns, efforts to control the spread of the disease, patient contacts, information flow, testing procedures and protection of staff.

**Results:**

GPs gave high ratings to their self-confidence (7.3, 95% CI 7.1–7.5) and their efforts to control the spread of the disease (7.2, 95% CI 7.0–7.3). A decrease in the number of patient contacts (5.7, 95% CI 5.4–5.9), the perception of risk (5.3 95% CI 4.9–5.6), the provision of information to GPs (4.9, 95% CI 4.6–5.2), their testing of suspected cases (3.7, 95% CI 3.4–3.9) and their preparedness to face a pandemic (mean: 3.5; 95% CI 3.2–3.7) were rated as moderate. GPs gave low ratings to their ability to protect staff (2.2 95% CI 1.9–2.4). Differences were identified in all dimensions except protection of staff, which was consistently low in all surveyed GPs and countries.

**Conclusion:**

Although GPs in the different countries were confronted with the same pandemic, its impact on specific aspects differed. This partly reflected differences in health care systems and experience of recent pandemics. However, it also showed that the development of structured care plans in case of future infectious diseases requires the early involvement of primary care representatives.

## Introduction

Following the reports of the first cases of COVID-19 in late 2019, the World Health Organization declared COVID-19 to be a Public Health Emergency of International Concern on 30^th^ January 2020, and a pandemic on 11^th^ March 2020 (1). Two years later, on 11^th^ March 2022, over 450 million cases of COVID-19, and over 6 million deaths, had been reported to the World Health Organization worldwide (2).

The pandemic has put unprecedented strain on healthcare systems, and, amongst other aspects, has had a profound effect on healthcare staff, healthcare delivery and utilization (3, 4). However, the focus of research has been on evaluating the pandemic's impact on hospital care and inpatient staff. Relatively little attention has been paid to its impact on primary care and on primary healthcare providers (5, 6).

The importance of primary care in dealing with this—or any other—pandemic, cannot be understated. It is the primary healthcare setting that is frequently the patient's first point of contact with the healthcare system (7), and primary care providers play a substantial role in identifying, managing and monitoring cases (8, 9).

Existing evidence suggests that the pandemic's impact on primary care has been considerable and multi-faceted (6). General Practitioners (GPs) in Italy, for example, have reported a high prevalence of adverse mental health outcomes, such as anxiety, depression and burnout (5). Changes have also occurred in the delivery of primary care, with GPs in many countries shifting from face-to-face consultations to remote telehealth consultations (e.g., phone or videoconferencing) (5, 6, 8). Care has also tended to focus less than usual on chronic care (10), with diabetes, COPD, and hypertension being the most impacted conditions (11). Preventive healthcare, and cancer screening in particular, were also affected considerably (12). These changes are ongoing and are expected to have a negative long-term influence on health outcomes. Primary care providers will continue to play an important role in managing future waves of the pandemic, and in providing care to patients with long COVID and other conditions that tend to be aggravated in pandemics, such as anxiety disorders.

The aim of our study was to investigate the experiences of GPs in seven countries (Australia, Austria, Germany, Hungary, Italy, Slovenia, and Switzerland), in the early phases of the COVID-19 pandemic (13). We focused on GPs' preparedness to face a pandemic, their self-confidence and concerns, efforts to control the spread of the disease, patient contacts, information flow, testing procedures and on how well they felt their staff were protected.

## Methods

This manuscript was prepared in accordance with the CHERRIES criteria (14). COVI-Prim-*International* is part of the COVI-Prim project, which is described in detail elsewhere (13, 15). Briefly, GPs in seven different countries (Australia, Austria, Germany, Hungary, Italy, Slovenia, and Switzerland) were invited to answer a basic questionnaire. In two countries (Austria and Germany) further questionnaires were sent to participating GPs at regular intervals (16).

We first analyzed the baseline questionnaire that was distributed as part of the COVI-Prim-*International* project [48 closed items, eight dimensions: (1) self-confidence, (2) efforts to control the spread of the disease, (3) decrease in number of patient contacts, (4) perception of risk, (5) provision of information to GP, (6) testing suspected cases, (7) preparedness for a pandemic, (8) protection of staff; factor scores ranged from 0 to 10]. The questionnaire was transferred to LimeSurvey^®^ (Austria, Germany, Hungary, Italy, Slovenia, and Switzerland) or SurveyMonkey^®^ (Australia). Invitations to GPs to respond to the questionnaire were sent out based on the mailing lists of participating universities and local GP associations. As the lists probably overlapped, it is not possible to know precisely how many GPs were contacted and hence to calculate a response rate. Participants were first informed about the length of the survey, the investigators, and the purpose of the study. After the survey had been completed, all data on the online platform were stored in SPSS files. GPs received no financial incentive to participate. Surveys started at different time points in the participating countries. In most countries, GPs first began to answer the survey when the number of patients testing positively had begun to decrease. Completion rates ranged from 63.3% in Slovenia to 91.7% in Australia. The median time required to answer the questionnaire ranged from 11.0 min in Australia (interquartile range: 7.6–15.1) to 17.3 (IQR: 12.0–22.5) in Italy. Further details on completion rates and the time required to answer the questionnaire are reported elsewhere (13). In order to examine comparable situations in different countries, periods of time were chosen during which infection dynamics were similar. Since in some countries the survey did not commence until after the peak of the first wave of infections, we only included GPs that had answered the baseline questionnaire from that point onwards. The first day considered in this analysis was therefore defined as the day on which the number of new infections first fell below 2/3 of the maximum number of new infections observed during the first wave. GPs that answered the survey after April 3rd in Australia, April 4th in Austria, April 9th in Switzerland, April 11th in Slovenia, April 13th in Germany, April 15th in Italy and after May 3rd in Hungary were therefore included.

### Statistics

Baseline characteristics are presented as mean ±SD or median (min—max), as appropriate. Categorical variables are provided as absolute numbers and percentages. In the main analysis, environmental variables (country of survey; size of town in which practice was located: <5,000/5,000 to <20,000/20,000 to <100,000/≥100,000) that may have influenced the responses, and the role of the GP within the practice (employed vs. owner), were analyzed using General Linear Models. The main effects and all two-way interactions were analyzed. Bonferroni correction was used to take account of multiple testing. Estimated means and 95% confidence intervals were used to present the results. For a better understanding of the results, responses to the items on each scale were also presented. In this presentation, the response categories “yes” and “yes, probably” and the response categories “no, probably not” and “no” were combined. No statistical correction was carried out to adjust for non-representative samples.

### Ethics

The study protocol was approved by the Ethics Committee of Bond University, Australia (AS200424), Goethe University Frankfurt, Germany (ID 20-619). The study required no ethical approval under Austrian, Italian, Slovenian, Swiss, and Hungarian law.

## Results

### Demographics

The survey was filled out by 1,642 GPs from Australia (*n* = 120), Austria (*n* = 434), Germany (*n* = 583), Hungary (*n* = 190), Italy (*n* = 112), Slovenia (*n* = 86), and Switzerland (*n* = 117). Mean age of the GPs was 51.9 years (SD: 10.8). The majority of GPs were male (52.1%) and practiced in a city with fewer than 20,000 inhabitants (56.6%). All demographic characteristics are provided in Table 1.

**Table 1:** Baseline demographics.

	**All**
	***n* = 1,642**
**Country**	
Australia	120 (7.3%)
Austria	434 (26.4%)
Germany	583 (35.5%)
Hungary	190 (11.6%)
Italy	112 (6.8%)
Slovenia	86 (5.2%)
Switzerland	117 (7.1%)
**Age**	51.9 ± 10.8
**Sex**	
Male	855 (52.1%)
Female	780 (47.5%)
Other	7 (0.4%)
**Size of town of practice**	
<5,000	454 (27.6%)
5,000– <20,000	474 (28.9%)
20,000– <100,000	270 (16.4%)
≥100,000	443 (27.0%)
Missing	1 (0.1%)
**Position in the practice**	
Employed	356 (21.7%)
Owner	1,284 (78.2%)
Missing	2 (0.1%)
**Year practice was set up**	Median: 2003
	IQR: 1992–2012

### Overall results

GPs gave high ratings to their self-confidence (7.3, 95% CI 7.1–7.5) and their efforts to control the spread of the disease (7.2, 95% CI 7.0–7.3). The decrease in the number of patient contacts (5.7, 95% CI 5.4–5.9), GPs' perception of risk (5.3 95% CI 4.9–5.6), the provision of information to GPs (4.9, 95% CI 4.6–5.2), testing of suspected cases (3.7, 95% CI 3.4–3.9) and their preparedness for a pandemic (mean: 3.5; 95% CI 3.2–3.7) were rated as moderate. GPs gave low ratings to their efforts to protect staff (2.2 95% CI 1.9–2.4).

### Differences between countries

#### Self-confidence

Austrian GPs rated their self-confidence (8.0, 95% CI 7.5–8.5) significantly higher than Hungarian (6.7, 95% CI 6.3–7.2, *p* = 0.043) and Italian GPs (6.3, 95% CI 5.6–7.1, *p* = 0.036). More Austrian GPs were further convinced they knew how to provide the best possible care to their patients during the pandemic (AT: 88.2%, HU: 76.1%, IT: 69.7%) (Supplementary Table S1).

#### Efforts to control the spread of the disease

Slovenian GPs rated their efforts to control the spread of the virus in the practice (8.0, 95% CI 7.5–8.6) more highly than Hungarian GPs (6.5, 95% CI 6.2–6.9, *p* = 0.003). The most pronounced differences between Slovenian and Hungarian GPs were observed in the number of GPs contacting patients that were quarantined at home in order to monitor the progression of the disease (SI: 72.2%, HU: 2.0%), and in the number of GPs that preferred to treat patients with mild illnesses that were not linked to suspected cases of COVID-19 by phone or online (SI: 98.4%, HU: 65.4%).

#### Decrease in number of patient contacts

German GPs estimated the decrease in the number of patient contacts (7.2, 95% CI 6.9–7.4) to have been significantly higher than did Australian (5.8, 95% CI 5.2–6.3, *p* < 0.001), Hungarian (5.6, 95% CI 5.0 to 6.1, *p* < 0.001), Italian (4.6, 95% CI 3.7–5.4, *p* < 0.001) and Slovenian (4.3, 95% CI 3.4–5.1, *p* < 0.001) GPs. Furthermore, Austrian GPs reported the decrease in the number of patient contacts (6.3, 95% CI 5.7–6.9, *p* = 0.007) was significantly higher than did Slovenian GPs.

#### Perception of risk

**Figure 1:**
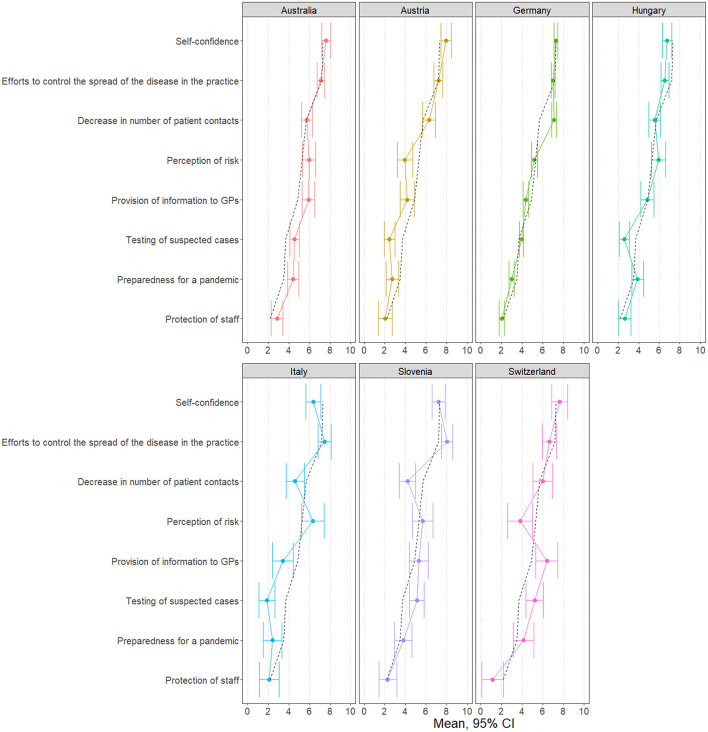
Scale values for each country (dashed black line: overall mean).

Austrian GPs perceived the risks they faced (3.9, 95% CI 3.2–4.7) to be considerably lower than did Hungarian (6.0, 95% CI 5.3–6.6, *p* = 0.010) Australian (6.0, 95% CI 5.3–6.7, *p* = 0.006) and Italian (6.4, 95% CI 5.3–7.5, *p* = 0.023) GPs (Table 2, Figure 1). On an item level, the biggest difference between Austrian and Australian GPs was in their perception of their employees' concerns about catching COVID-19 from patients (AU: 72.1 vs. AT: 37.2%). As far as the conflict between wanting to care for their patients but at the same time not wishing to endanger their families was concerned, the biggest differences were between Austrian, and Hungarian and Italian GPs (AT: 46.8, HU 68.4, IT: 70.1%).

**Table 2:** Mean and 95% CI for each factor used in the evaluation of the pandemic for participating countries.

	**Country**						
	**Australia**	**Austria**	**Germany**	**Hungary**	**Italy**	**Slovenia**	**Switzerland**
	**A**	**B**	**C**	**D**	**E**	**F**	**G**
Self confidence	7.7 (7.3–8.1)	**8.0 (7.5**–**8.5)**^D^, ^E^	7.3 (7.1–7.5)	**6.7 (6.3**–**7.2)**^B^	**6.3 (5.6–7.1)** ^B^	7.2 (6.6–7.9)	7.6 (6.8–8.4)
Efforts to control the spread of the disease in the practice	7.2 (6.8–7.5)	7.2 (6.8–7.6)	7.0 (6.8–7.1)	**6.5 (6.2** to **6.9)**^F^	7.5 (6.9–8.1)	**8.0 (7.5**–**8.6)**^D^	6.7 (6.0–7.3)
Decrease in number of patient contacts	**5.8 (5.2**–**6.3)**^C^	**6.3 (5.7**–**6.9)**^F^	**7.2 (6.9**–**7.4)**^A^, ^D^, ^E^, ^F^	**5.6 (5.0**–**6.1)**^C^	**4.6 (3.7**–**5.4)**^C^	**4.3 (3.4**–**5.1)**^B^, ^C^	6.0 (5.0–6.9)
Perception of risk	**6.0 (5.3**–**6.7)**^B^	**3.9 (3.2**–**4.7)**^A^, ^D^, ^E^	5.1 (4.8–5.4)	**6.0 (5.3**–**6.6)**^B^	**6.4 (5.3**–**7.5)**^B^	5.6 (4.6–6.6)	3.9 (2.7–5.1)
Provision of information to GPs	**5.9 (5.3**–**6.5)**^B^, ^C^, ^E^	**4.2 (3.5**–**4.9)**^A^	**4.5 (4.2**–**4.7)**^A^	4.8 (4.2–5.5)	**3.3 (2.3**–**4.3)**^A^, ^G^	5.4 (4.5–6.3)	**6.4 (5.3**–**7.4)**^E^
Testing of suspected cases	**4.5 (4.0**–**5.0)**^B^, ^D^, ^E^	**2.4 (1.9**–**3.0)**^A^, ^C^, ^F^, ^G^	**3.9 (3.7**–**4.1)**^B^, ^D^, ^E^	**2.6 (2.1**–**3.1)**^A^, ^C^, ^F^, ^G^	**1.9 (1.1**–**2.7)**^A^, ^C^, ^F^, ^G^	**5.1 (4.4**–**5.8)**^B^, ^D^, ^E^	**5.2 (4.4**–**6.1)**^B^, ^D^, ^E^
Preparedness for a pandemic	**4.3 (3.8**–**4.9)**^B^, ^C^, ^E^	**2.7 (2.1**–**3.4)**^A^	**3.0 (2.8**–**3.3)**^A^	3.9 (3.3–4.5)	**2.4 (1.5**–**3.3)**^A^	3.8 (3.0–4.6)	4.1 (3.1–5.1)
Protection of staff	2.9 (2.3–3.5)	2.0 (1.4–2.7)	2.0 (1.7–2.2)	2.7 (2.1–3.3)	2.2 (1.2–3.1)	2.2 (1.4–3.1)	1.2 (0.1–2.2)

A *post-hoc* comparison to Australia, *p* < 0.05 (Bonferroni corrected).

B *post-hoc* comparison to Austria, *p* < 0.05 (Bonferroni corrected).

C *post-hoc* comparison to Germany, *p* < 0.05 (Bonferroni corrected).

D *post-hoc* comparison to Hungary, *p* < 0.05 (Bonferroni corrected).

E *post-hoc* comparison to Italy, *p* < 0.05 (Bonferroni corrected).

F *post-hoc* comparison to Slovenia, *p* < 0.05 (Bonferroni corrected).

G *post-hoc* comparison to Switzerland, *p* < 0.05 (Bonferroni corrected).

Significant differences are in bold (Scale values range from 0 to 10).

#### Provision of information to GPs

Swiss GPs rated the provision of information to GPs (6.4, 95% CI 5.3–7.4) more highly than Italian GPs (3.3, 95% CI 2.3–4.3, *p* = 0.006). The biggest difference in their evaluations was in whether the guidelines on how to deal with suspected cases of COVID-19 were sufficiently detailed (CH: 87.3 vs. IT: 32.4%). Australian GPs rated the provision of information to GPs (5.9, 95% CI 5.3–6.5) more highly than Italian (*p* = 0.001), Austrian (4.2, 95% CI 3.5–4.9, *p* = 0.018) and German (4.5, 95% CI 4.2–4.7, *p* = 0.002) GPs. Between Australian and Italian GPs, the biggest difference in the evaluation was whether they had received guidelines on how to deal with suspected cases of COVID-19 in a timely manner (AU:84.8 vs. IT: 33.8%).

#### Testing of suspected cases

Differences in the evaluation of the effort expended on testing suspected cases varied substantially between countries. Italian (1.9, 95% CI 1.1–2.7), Austrian (2.4, 95% CI 1.9–3.0) and Hungarian (2.6, 95% CI 2.1–2.7). GPs rated the effort expended on testing suspected cases as significantly lower (*p* < 0.001) than did German (3.9, 95% CI 3.7–4.1), Australian (4.5, 95% CI 4.0–5.0), Slovenian (5.1, 95% CI 4.4–5.8) and Swiss (5.2, 95% CI 4.4–6.1) GPs. On an item level, 92.6% of Italian and 82.7% of Austrian GPs but only 12.9% of Slovenian GPs thought the number of tests that were conducted was insufficient. Furthermore, 10.1% of Austrian and 5.9% of Italian GPs but 81.0% of the Slovenian GPs said that they had adequate access to tests from the beginning of the COVID-19 pandemic.

#### Preparedness for a pandemic

Australian GPs rated their preparedness for a pandemic (4.3, 95% CI 3.8–4.9) higher than German (3.0, 95% CI 2.8–3.3, *p* = 0.003) Austrian (2.7, 95% CI 2.1–3.4, *p* = 0.020), and Italian GPs (2.4, 95% CI 1.5–3.3, *p* = 0.049). Big differences between Australian and German, Austrian and Italian GPs were found in how they rated the preparedness of their practices to face a pandemic (AU: 43.8, AT: 23.1, DE: 23.3, IT: 27.3%), and whether they had sufficient information on how much protective equipment they would need (AU: 26.8, AT: 8.0, DE: 9.3, IT: 14.9%).

#### Protection of staff

No differences between countries were found in GPs' effort to protect staff.

## Discussion

### Overall results

Our 2020 survey of over 1,600 general practitioners from seven countries revealed that GPs generally had considerable self-confidence and went to great efforts to control the spread of the COVID-19 pandemic. In the first stages, GPs in Europe and Australia were not well prepared to face a pandemic, had to confront a fall in the number of patient contacts and had to make do with unsatisfactory testing procedures for suspected cases. GPs regarded the risks of the pandemic as moderate, but only received limited information from health care authorities on how to deal with suspected cases of COVID-19. Between the various countries, the responses of the GPs differed in all dimensions except for the protection of staff, which was consistently low. Differences are understandable as GPs have to deal with the specific challenges of acting within the primary health care system of their own country (7, 17–25).

### Preparedness to face a pandemic

In accordance with other previously published papers, the highest scores in preparedness to face a pandemic were observed in Australia (22), and the lowest scores in Italy (20). While in Australia, the role of GPs during a pandemic has been discussed in scientific papers for a long time (26, 27), the topic in central Europe was only a minor issue before the outbreak of COVID-19. As early as 2006, Shaw (28) said it was important that Australian GPs were in a position to continue working effectively during a pandemic, adding that to be able to do this, it was essential that they receive appropriate education, training and equipment. Furthermore, Australian experts discussed how important it is that GPs participate in surveillance systems to identify clusters, and are involved in adapting the system in preparation for a pandemic in good time (29, 30). In Europe, the role GPs might play in a pandemic was discussed in several countries [e.g., the Netherlands (31), Great Britain (32), Germany (33) and Hungry (34)]. However, this research activity in Europe was limited to isolated publications. As Xiao et al. pointed out, not only the appropriate education and training for pandemic situations, but also the general education for GPs is in crucial aspect of preparedness (35).

Pandemic preparedness is a global issue. One year before the outbreak of COVID-19, Gupta et al. recognized that many countries were not adequately prepared to face a pandemic (36). More than 50% of countries scored inadequately in terms of almost 90% of the indicators used to gauge preparedness for outbreaks of infectious diseases. Oppenheimer et al. also found that many countries were not prepared for a pandemic (37). Furthermore, both studies found that an association existed between preparedness and the economic strength of a country. Therefore, countries in Europe and North America were most prepared and independent of region a higher GDP per capita, more public health expenditures as a percentage of GDP and higher density of skilled health professionals per 10.000 persons were associated with a better preparedness (36, 37).

### Self-confidence and perception of risk

Self-confidence, or the belief in one's ability to successfully accomplish specific goals, and perception of risk, both play an important role in GPs' professional and private lives. We could observe differences between GPs in different countries in terms of both risk perception and self-confidence. Similar results for COVID-19 risk perceptions have already been reported for overall populations, and among health care workers and GPs (38, 39). Furthermore, it is not only the overall level of risk perception that differs between countries, but also the predictors of risk perceptions. While political ideology was an important predictor of COVID-19 risk perceptions in South Korea and the United States, social amplification was significantly more important in Australia, Germany, Spain, Japan, Sweden and the United Kingdom (39, 40). It is also interesting that COVID-19 risk perceptions are not associated with local epidemic severity (38). Risk perceptions are, however, an important predictor of self-confidence (40). In GPs and health care workers, it was found that a higher level of confidence is associated with lower levels of emotional exhaustion, anxiety in general, COVID-19 anxiety, and concerns about one's family, as well as with higher levels of self-perceived preparedness (41–43). On the other hand, such aspects as social support and quality of sleep, and pre-crisis education and training programmes, can also increase self-confidence (44, 45).

### Decrease in numbers

The provision of primary healthcare services decreased in all countries, and especially in Germany and Austria, where GPs said that many patients avoided coming to the practice. In Switzerland and Australia, the number of patient contacts decreased the least. Drawing on data from over 80 million visits in 2019 and 2020, the INTernational ConsoRtium of Primary Care BIg Data Researchers (INTRePID) confirmed that a fall in patient contacts was a global phenomenon. Nevertheless, primary care physician consultations remained stable in Australia (19). One longitudinal observational study from Germany comparing the pre- COVID period with April–July 2020 described a significant decrease in GP consultations per week for conditions relating to the total and upper gastrointestinal tract, vertigo, spinal disorders, general fatigue and weakness, as well as in 12 further services (house calls, stool tests, referrals to a specialist, check-up 35, urine analysis, pain therapy, skin cancer screening, electrocardiograms, blood tests, pulmonary function tests, sonography, and wound management) (25). A decline in practice visits was accompanied by a clear shift to telemedicine appointments, as highlighted in a narrative review published by Kichloo et al. (46). The number of telemedicine appointments was particularly high at the beginning of the pandemic, but decreased later (16).

### Provision of information

GPs in Switzerland and Australia rated the amount of information they had been provided with more highly than other countries, which is probably because they received guidelines on how to deal with suspected COVID-19 cases earlier (Supplementary Table S1). As in Switzerland and Australia, Slovenian GPs also appear to have enjoyed a more productive exchange of information with health authorities than other countries. Nevertheless, the majority of GPs in our study said that information was available on public media before it was officially provided by official institutions such as health insurers. The problem of inadequate communication with the health authorities was described in an Australian publication as soon as 2015 (27), and further confirmed by Rawaf et al., who concluded from a worldwide survey that primary care professionals were poorly informed by policymakers (47). As insufficient information was available from public stakeholders, many GPs creatively established and used regional networks to share information during the first phase of the COVID-19 pandemic (13, 18, 22, 24, 47).

### Testing of suspected cases

The availability of diagnostic COVID-19 testing varied substantially across countries. Our analyses revealed that GPs from Italy, Hungary and Austria said that in the early stages, access to laboratory testing for COVID-19 in GPs practices was inadequate and rarely carried out. These GPs increasingly demanded that they should be permitted to decide who should be tested and who should not (see Supplementary Table S1). Since there are differences between countries in the number of tests per million inhabitants, it is to be expected that the GPs assessments would vary (48). The number of tests not only differed between countries, but also developed very differently over time (48). It should be kept in mind that our survey was conducted before routine diagnostic COVID-19 testing was available, i.e., only PCR testing was possible and rapid antigen screening tests did not exist. Circumstances have therefore changed substantially, and there appears to no longer be any shortage of test kits in the countries under review.

### Protection of staff

GPs in our survey complained that they could not protect their staff, but such complaints were not only heard from GPs, with the British Medical Association, for example, also warning that doctors were at “considerable” risk due to a lack of personal protective equipment (49, 50). Furthermore, the Global Forum on Universal Health Coverage and Primary Health Care, which represents 29 countries, also said they were insufficiently equipped to provide care to protect staff (47). In contrast, a survey of 361 Chinese GPs (of whom 54 worked in hospitals) (43) found that GPs were overall well-equipped and supported during the outbreak of the COVID-19 pandemic, particularly in large Chinese cities. In order to deal with the virus, nearly all clinics provided training and seminars, and gave talks, not only to healthcare workers but also to the general public. Almost all GPs provided information to their patients during consultations, on social media and the telephone, and by means of posters and leaflets in clinics. However, despite all these efforts, only 15% of hospital GPs thought their clinic provided sufficient support to protect staff.

### Strengths and limitations

Our study has some limitations. Firstly, the questionnaire was developed in a very short time in order that it could be answered when the situation was most acute. Even though we tried to include all relevant topics, some issues may therefore have been missed. Secondly, we could not calculate the response rate because a systematic area-wide survey was not possible in the time frame we permitted ourselves. Nevertheless, the number of responses far exceeded our expectations, especially considering the difficulties that are usually encountered in recruiting GPs for research. Thirdly, as the recruitment process was conducted through regional networks and professional associations, the participants may not have been representative of GPs as a whole. Fourthly, we know that online surveys are not as suitable for the collection of in-depth information as interviews. Fifth, this cross-sectional survey was carried out in the first half of 2020, since when many changes have occurred in pandemic management. For this reason, we also carried out a longitudinal survey in Germany and Austria, which revealed that physicians in primary care have adapted quickly to new situations and have gained experience in telemedicine, enabling them to overcome changes in the delivery of routine health care (16). Despite involving seven countries, our study was not truly representative. However, the PRICOV-19 study that is being carried out in 37 European countries and Israel is currently ongoing and should provide more information on how GP practices have functioned during the COVID-19 pandemic on a European level (51). One further limitation is that our survey was only carried out among GPs and did not involve other team members from a primary care setting. Nevertheless, the study succeeded in providing an insight into the challenges of providing care in the early stages of the pandemic in a wide range of countries, and it identifies substantial differences between them. A further limitation is that sample sizes vary between countries. These unbalanced sample sizes may have an impact on the results.

Last but not least, the questionnaire was used in different languages. It cannot therefore be ruled out that differences between countries reflect discrepancies in the translation. We used differential item functioning (DIF) to investigate the extent of any discrepancies. This analysis investigates whether GPs from different groups (e.g., countries; pairwise comparisons) that are being assessed using the same scales (e.g., self-confidence) answer individual items in a similar way. The analysis revealed that on two scales (self-confidence, preparedness) and 16 further items, no DIF could be found, while only one DIF was found in 6 out of 39 investigated items. Since the number of DIFs is comparable in countries where the same language is spoken (e.g., Austria – Germany: *n* = 4; Switzerland- Germany: *n* = 4) and countries using different languages (e.g., Australia–Germany: *n* = 3), the DIFs are assumed to be due to aspects other than language (e.g., differences in primary health care systems). The highest number of DIFs could be observed in a comparison between Germany and Slovenia (*n* = 8) (Supplementary Table S2).

## Conclusion

Although general practitioners in different countries were confronted with the same pandemic, its impact differed in a number of aspects (e.g., self-confidence, perception of risk). To some extent this may be because countries presumably had different levels of experiences regarding prior pandemics and therefore in how well-prepared they were to face them. This, in turn, may have been reflected in differences in the provision of information and equipment to GPs. Differences in health care systems between the countries may also have had an impact. Knowledge of these differences is important and should be shared in order to be better prepared for future pandemics. It is also essential that primary care representatives are involved in the preparation of structured care plans for future infectious diseases at an early stage.

**Question:** Are there differences regarding management and personal experience of general practitioners in seven countries at the beginning of the COVID-19 pandemics?

**Finding:** Despite facing the same pandemic, there was substantial variation in almost all evaluated dimensions, for which differences in health care systems and experiences with recent pandemics may be responsible.

**Meaning:** GPs should be involved in developing a coordinated strategy to deal with pandemics, which should be communicated to affected health care institutions and populations as early as possible.

## Reproducible research statement

*Study protocol:* Detailed information is provided in the Supplement of the Siebenhofer et al. (13). *Data set:* Available from AA, PhD (alexander.avian@medunigraz.at).

## Data availability statement

The raw data supporting the conclusions of this article will be made available by the authors, without undue reservation.

## Ethics statement

The studies involving human participants were reviewed and approved by Ethics Committee of Bond University, Australia (AS200424). Ethics Committee of Goethe University Frankfurt, Germany (ID 20-619). Written informed consent for participation was not required for this study in accordance with the national legislation and the institutional requirements.

## Author contributions

Conceptualization: ASi, AA, DS-S, KM, SH, HB, MF, ASc, and MG. Data curation: SH and AA. Formal analysis and visualization: AA. Investigation: AA, SH, ASc, AT, EZ, AM, GP, SS, KJ, DS-S, KM, HB, MF, ASi, and MG. Methodology: AA, HB, MF, and ASi. Project administration: DS-S, KM, SH, MF, and ASi. Resources: ASi and KM. Supervision: ASi and MF. Writing—original draft: AA, ASc, ASi, and MG. Writing—review and editing: AT, EZ, AM, GP, SS, KJ, DS-S, KM, AA, SH, HB, MF, ASi, and MG. All authors contributed to the article and approved the submitted version.

## Conflict of interest

The authors declare that the research was conducted in the absence of any commercial or financial relationships that could be construed as a potential conflict of interest.

## Publisher's note

All claims expressed in this article are solely those of the authors and do not necessarily represent those of their affiliated organizations, or those of the publisher, the editors and the reviewers. Any product that may be evaluated in this article, or claim that may be made by its manufacturer, is not guaranteed or endorsed by the publisher.
