# Supplementary material for: COVI-Prim international: Similarities and discrepancies in the way general practices from seven different countries coped with the COVID-19 pandemic
Source: Front Public Health. 2022 Dec 6;10:1072515. doi: 10.3389/fpubh.2022.1072515 (PMC9806865; doi:10.3389/fpubh.2022.1072515)
Supplement: Supplementary file 1 [file Table_1.DOCX]

**Supplement**

Suppl. Table 1: Percentage of GPs answering “yes” or “probably yes” for each country. Red cells: observed percentage more than 5% above average; green cells: observed percentage more than 5% below average

| **Self-confidence** | AU | AT | DE | HU | IT | SI | CH |
| --- | --- | --- | --- | --- | --- | --- | --- |
| I am convinced that I know enough to provide optimal care for my patients during the pandemic. | 83.9% | 88.2% | 79.0% | 76.1% | 69.7% | 77.4% | 87.4% |
| I know what to do in case of a suspected case of COVID-19. | 100.0% | 99.1% | 99.6% | 98.7% | 97.1% | 100.0% | 100.0% |
| When looking after patients that have been infected with COVID-19. I am sometimes unsure that I am doing everything right. | 31.2% | 26.6% | 33.4% | 36.4% | 58.5% | 50.0% | 22.2% |

| **Efforts to control the spread of the disease** | AU | AT | DE | HU | IT | SI | CH |
| --- | --- | --- | --- | --- | --- | --- | --- |
| I do not currently treat patients with mild illnesses that are not linked to suspected cases of COVID-19 in my practice, and attend to them by phone or online. | 58.9% | 88.9% | 83.9% | 65.4% | 89.4% | 98.4% | 42.7% |
| If possible, I, or one of my employees, tries to gain enough information from patients by phone in order to know whether we are dealing with a suspected case of COVID-19. | 96.4% | 97.6% | 99.0% | 98.7% | 97.1% | 98.4% | 91.3% |
| I use various digital channels (e.g. e-mail, WhatsApp) to share information with my colleagues so that we can support each other in the current situation. | 84.8% | 81.5% | 76.7% | 87.7% | 91.2% | 92.1% | 75.0% |
| I have taken precautions to ensure that suspected cases do not come into contact with other patients in my practice (e.g. separate waiting rooms, appointments at different times). | 98.2% | 97.6% | 96.3% | 96.1% | 97.0% | 95.2% | 97.1% |
| I contact patients that are quarantined at home in order to monitor the progression of the disease. | 39.3% | 57.9% | 73.8% | 2.0% | 86.6% | 72.2% | 72.5% |
| I avoid touching patients when examining them. | 89.3% | 43.1% | 38.1% | 40.9% | 47.8% | 50.0% | 26.5% |
| Before a patient enters my practice, he or she is screened for possible symptoms (e.g. temperature measurement). | 86.5% | 59.9% | 55.4% | 88.5% | 72.1% | 95.2% | 36.9% |

| **Decrease in number of patient contacts** | AU | AT | DE | HU | IT | SI | CH |
| --- | --- | --- | --- | --- | --- | --- | --- |
| I have less to do at the moment because many patients are not currently coming to the practice. | 51.8% | 76.0% | 76.1% | 51.9% | 44.1% | 15.9% | 41.2% |
| I have to look after more patients because other health care services (specialists. hospitals) are less available. | 27.7% | 49.0% | 25.9% | 41.7% | 58.8% | 60.7% | 16.3% |
| I have less contact to patients as a result of the pandemic. | 41.4% | 95.9% | 93.7% | 75.5% | 88.1% | 80.6% | 40.4% |
| I am currently treating patients that I would normally refer to specialists or to hospital. | 41.1% | 65.0% | 42.2% | 62.3% | 79.1% | 58.3% | 14.7% |

| **Perception of risk** | AU | AT | DE | HU | IT | SI | CH |
| --- | --- | --- | --- | --- | --- | --- | --- |
| I am worried that people I live with could catch COVID-19 from me. | 71.4% | 45.6% | 56.4% | 67.1% | 61.2% | 74.2% | 34.6% |
| I am afraid that I will catch COVID-19 from a patient. | 56.3% | 26.1% | 33.9% | 41.0% | 44.1% | 41.3% | 14.6% |
| It causes me concern that I want to care for my patients but at the same time do not want to endanger my family. | 76.8% | 46.8% | 49.3% | 68.4% | 70.1% | 73.0% | 27.2% |
| I am worried that I may unknowingly infect my patients. | 61.6% | 45.6% | 54.3% | 65.2% | 58.8% | 61.9% | 25.0% |
| My employees are worried about catching COVID-19 from patients. | 72.1% | 37.2% | 53.2% | 53.6% | 48.5% | 74.6% | 21.8% |

| **Provision of information to GPs** | AU | AT | DE | HU | IT | SI | CH |
| --- | --- | --- | --- | --- | --- | --- | --- |
| I received guidelines on how to deal with suspected cases of COVID-19 in good time. | 84.8% | 57.9% | 60.2% | 58.3% | 33.8% | 73.0% | 76.7% |
| The guidelines on how to deal with suspected cases of COVID-19 were sufficiently detailed. | 77.5% | 60.5% | 62.1% | 67.7% | 32.4% | 69.8% | 87.3% |
| At the beginning of the COVID-19 pandemic, I received sufficient information from public bodies | 56.3% | 33.9% | 28.7% | 30.3% | 20.6% | 58.1% | 57.7% |
| At the beginning of the COVID-19 pandemic, I had sufficient information on how to deal with suspected cases. | 66.1% | 52.6% | 45.4% | 48.4% | 34.3% | 71.0% | 66.3% |
| My employees and I were easily able to contact the responsible health care authorities. | 57.1% | 44.3% | 33.3% | 61.0% | 28.4% | 68.3% | 57.4% |
| Important information was available to patients on public media sooner than it was officially provided to family practitioners in information letters from the responsible institutions (e.g. health insurance funds). | 56.3% | 73.7% | 69.3% | 62.5% | 66.7% | 62.3% | 55.4% |

| **Testing suspected cases** | AU | AT | DE | HU | IT | SI | CH |
| --- | --- | --- | --- | --- | --- | --- | --- |
| Too little testing is being done. | 41.1% | 82.7% | 64.1% | 74.5% | 92.6% | 12.9% | 32.0% |
| At the beginning of the COVID-19 pandemic I had adequate access to tests (either conducted them myself. or could arrange them). | 52.7% | 10.1% | 39.0% | 25.8% | 5.9% | 81.0% | 38.8% |
| It would be best if all suspected cases of COVID-19 went directly to hospital so that I could look after the rest of the patients. | 34.8% | 26.0% | 11.1% | 42.8% | 29.4% | 46.0% | 19.8% |
| Separate hotlines should be available to enable medical personnel to arrange tests for patients. | 73.0% | 94.3% | 80.3% | 83.2% | 98.5% | 80.8% | 65.3% |
| We family practitioners should be able to decide who gets tested and who doesn't. | 85.7% | 96.1% | 92.8% | 93.6% | 97.1% | 77.0% | 69.9% |

| **Preparedness for a pandemic** | AU | AT | DE | HU | IT | SI | CH |
| --- | --- | --- | --- | --- | --- | --- | --- |
| At the beginning of the COVID-19 pandemic, I had enough protective equipment on hand. | 23.2% | 13.9% | 12.8% | 16.7% | 10.4% | 25.4% | 24.0% |
| My practice was well prepared for the COVID-19 pandemic. | 43.8% | 23.1% | 23.3% | 22.1% | 27.3% | 50.8% | 43.1% |
| At the beginning of the COVID-19 pandemic, I knew where I could get hold of protective equipment. | 24.1% | 20.6% | 22.9% | 34.6% | 21.2% | 41.9% | 43.7% |
| At the beginning of the COVID-19 pandemic, I had sufficient information on how much equipment I need. | 26.8% | 8.0% | 9.3% | 25.8% | 14.9% | 39.7% | 18.4% |
| Currently I have enough personal protective equipment. | 61.6% | 58.2% | 63.5% | 68.4% | 55.9% | 80.3% | 89.4% |

| **Protection of staff** | AU | AT | DE | HU | IT | SI | CH |
| --- | --- | --- | --- | --- | --- | --- | --- |
| I have had to send employees home because we had too little protective equipment. | 5.4% | 15.2% | 12.0% | 9.2% | 30.3% | 10.9% | 1.9% |
| Some employees in my practice have ceased working since the outbreak of the COVID-19 pandemic because they belong to a vulnerable group (e.g. pregnant women, older employees). | 46.4% | 13.6% | 18.5% | 19.9% | 19.4% | 45.2% | 13.5% |
| I found it difficult to provide adequate information to my practice team without worrying them. | 38.4% | 14.5% | 22.1% | 22.1% | 13.2% | 18.0% | 12.5% |

Suppl. Table 2: DIF – analysis. Significant DIF compared to Germany (p-values are shown). Germany was chosen as reference because the sample size there was the largest.

| Scale | Item | Austria | Australia | Switzerland | Hungary | Italy | Slovenia |
| --- | --- | --- | --- | --- | --- | --- | --- |
| Self-Confidence | 1 | 0.610 | 0.610 | 0.610 | 1.000 | 1.000 | 1.000 |
| Self-Confidence | 2 | 1.000 | 1.000 | 1.000 | 1.000 | 1.000 | 1.000 |
| Self-Confidence | 3 | 1.000 | 1.000 | 1.000 | 1.000 | 1.000 | 1.000 |
| Spread | 1 | 1.000 | 1.000 | 1.000 | 1.000 | 1.000 | 1.000 |
| Spread | 2 | 1.000 | 1.000 | 1.000 | 1.000 | 1.000 | 1.000 |
| Spread | 3 | 1.000 | 1.000 | 1.000 | 0.191 | 0.191 | 0.191 |
| Spread | 4 | 1.000 | 1.000 | 1.000 | 1.000 | 1.000 | 1.000 |
| Spread | 5 | 1.000 | 1.000 | 1.000 | 0.048 | 0.048 | 0.048 |
| Spread | 6 | 1.000 | 1.000 | 1.000 | 1.000 | 1.000 | 1.000 |
| Spread | 7 | 1.000 | 1.000 | 1.000 | <0.001 | <0.001 | <0.001 |
| Patients | 1 | 0.308 | 0.084 | 0.084 | 1.000 | 1.000 | 0.062 |
| Patients | 2 | 0.033 | 0.027 | 0.027 | 1.000 | 0.273 | 0.009 |
| Patients | 3 | 1.000 | <0.001 | <0.001 | 0.029 | 0.095 | <0.001 |
| Patients | 4 | 0.426 | 1.000 | 1.000 | 0.741 | 1.000 | 0.020 |
| Risk Perception | 1 | 1.000 | 0.665 | 0.013 | 1.000 | 1.000 | 1.000 |
| Risk Perception | 2 | 0.697 | 0.279 | 1.000 | 1.000 | 1.000 | 0.353 |
| Risk Perception | 3 | 0.041 | 1.000 | 1.000 | 0.075 | 1.000 | 1.000 |
| Risk Perception | 4 | 0.034 | 1.000 | 0.439 | 1.000 | 0.162 | 1.000 |
| Risk Perception | 5 | 1.000 | <0.001 | 0.788 | 1.000 | 1.000 | 1.000 |
| Informtation | 1 | 1.000 | 1.000 | 1.000 | 0.804 | 0.727 | 0.016 |
| Informtation | 2 | 1.000 | 1.000 | 1.000 | 0.285 | 0.493 | 0.305 |
| Informtation | 3 | 1.000 | 1.000 | 1.000 | 0.961 | 1.000 | 0.802 |
| Informtation | 4 | 1.000 | 1.000 | 1.000 | 0.033 | 1.000 | 0.151 |
| Informtation | 5 | 1.000 | 1.000 | 1.000 | 0.005 | 0.573 | 0.017 |
| Informtation | 6 | 1.000 | 1.000 | 1.000 | 1.000 | 0.133 | 1.000 |
| Informtation | 7 | 1.000 | 1.000 | 1.000 | 1.000 | 0.132 | 1.000 |
| Testing | 1 | 0.049 | 1.000 | 1.000 | 0.018 | 0.018 | 1.000 |
| Testing | 2 | 1.000 | 1.000 | 1.000 | 1.000 | 1.000 | 1.000 |
| Testing | 3 | 0.669 | 1.000 | 1.000 | 1.000 | 1.000 | 1.000 |
| Testing | 4 | 1.000 | 1.000 | 0.034 | 1.000 | 1.000 | 1.000 |
| Testing | 5 | 0.482 | 1.000 | 1.000 | 1.000 | 1.000 | 1.000 |
| Preparedness | 1 | 1.000 | 1.000 | 1.000 | 1.000 | 1.000 | 1.000 |
| Preparedness | 2 | 0.168 | 1.000 | 1.000 | 1.000 | 1.000 | 0.367 |
| Preparedness | 3 | 1.000 | 0.060 | 1.000 | 1.000 | 0.123 | 0.067 |
| Preparedness | 4 | 0.100 | 1.000 | 1.000 | 1.000 | 0.331 | 1.000 |
| Preparedness | 5 | 0.100 | 0.220 | 1.000 | 1.000 | 1.000 | 1.000 |
| Staff | 1 | 1.000 | 1.000 | 1.000 | 0.084 | 1.000 | 0.041 |
| Staff | 2 | 0.546 | 0.375 | 0.346 | 0.593 | 0.189 | 1.000 |
| Staff | 3 | 1.000 | 1.000 | 1.000 | 1.000 | 0.524 | 0.709 |
