# Supplementary material for: COVI-Prim international: Similarities and discrepancies in the way general practices from seven different countries coped with the COVID-19 pandemic
Source: Front Public Health. 2022 Dec 6;10:1072515. doi: 10.3389/fpubh.2022.1072515 (PMC9806865; doi:10.3389/fpubh.2022.1072515)
Supplement: Supplementary file 2 [file Table_2.DOCX]

**Supporting information: Checklist for Reporting Results of Internet E-Surveys (CHERRIES)**

| 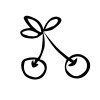 | **Checklist for Reporting Results of Internet E-Surveys (CHERRIES)** | |
| --- | --- | --- |
| ***Item Category*** | ***Checklist Item*** | ***Page*** |
| **Design** | Describe survey design | 4 |
| **IRB (Institutional Review Board) approval and informed consent process** | IRB approval | 5 |
|  | Informed consent | A-4, A-9 |
|  | Data protection | A-9 |
| **Development and pre-testing** | Development and testing | A-4 |
| **Recruitment process and description of the sample having access to the questionnaire** | Open survey versus closed survey | A-7 |
|  | Contact mode | A-8 |
|  | Advertising the survey | A-8 |
| **Survey administration** | Web/E-mail | 4, A-7 |
|  | Context | A-7 |
|  | Mandatory/voluntary | A-7 |
|  | Incentives | A-7 |
|  | Time/Date | A-7 |
|  | Randomization of items or questionnaires | A-8 |
|  | Adaptive questioning | A-8 |
|  | Number of Items | 5, A-4, A-5, A-6 |
|  | Number of screens (pages) | A-5, A-6 |
|  | Completeness check | A-8 |
|  | Review step | A-8 |
| **Response rates** | Unique site visitor | NA |
|  | View rate (Ratio of unique survey visitors/unique site visitors) | NA |
|  | Participation rate (Ratio of unique visitors who agreed to participate/unique first survey page visitors) | NA |
|  | Completion rate (Ratio of users who finished the survey/users who agreed to participate) | 4, A-9 |
| **Preventing multiple entries from the same individual** | Cookies used | A-8 |
|  | IP check | A-8 |
|  | Log file analysis | A-8 |
|  | Registration | NA |
| **Analysis** | Handling of incomplete questionnaires | A-6 |
|  | Questionnaires submitted with an atypical timestamp | A-8 |
|  | Statistical correction | A-9 |

**Supporting information: Study design^[[1]](#footnote-1)^**

COVI-Prim is an international project that plans to carry out regular surveys of GPs working in primary care during the COVID-19 pandemic in order to research their role in it, the specific challenges they face, and the strategies they have developed to deal with it (<https://allgemeinmedizin.medunigraz.at/news/>, <http://www.allgemeinmedizin.uni-frankfurt.de/forschung1/covi_prim.html>, <https://www.pmu.ac.at/allgemeinmedizin.html>). Potential deficiencies in care and possible obstacles such as a lack of stakeholder support are analyzed. An overview of the COVI-Prim project is provided in **S1 Table**.

*S1 Table . COVI-Prim overview.*

| Participating  Countries | Start of  project | Finalization /  Translation of  Questionnaire | Ethics  approval | Start of  the survey | End of  the survey | Baseline  survey | Longitudinal  survey | Sub - Project | | | |
| --- | --- | --- | --- | --- | --- | --- | --- | --- | --- | --- | --- |
|  |  |  |  |  |  |  |  | COVI-Prim-*Start* | COVI-Prim-*Flat* | COVI-Prim-*Long* | COVI-Prim-  *Hot topics* |
| Australia | 8^th^ April | 20^th^ April | Yes | 8^th^ May | 8^th^ August | x | x |  | X | X |  |
| Austria | 20^th^ March | 30^th^ March | N.A.* | 3^rd^ April | 29^th^ May | x | x | X | X | X | x |
| Germany | 20^th^ March | 30^th^ March | Yes | 3^rd^ April | 27^th^ May | x | x | X | X | X | x |
| Hungary | 7^th^ May | 3^rd^ June | N.A. | 5^th^ June | 2^nd^ July | x |  |  | X |  |  |
| Italy/German | 23^rd^ April | German | N.A. | 23^rd^ April | 6^th^ May | x |  |  | x |  | x |
| Slovenia | 29^th^ April | 15^th^ May | Yes | 1^st^ June | 6^th^ July | x |  |  | x |  |  |
| Switzerland | 15^th^ May | German/Italian (31^st^ May) | N.A. | 7^th^ July | 4^th^ August | x |  |  | x |  |  |

N.A. …Not applicable

* … the head of the local ethic committee stated, that according to the Austrian law, this study does not require an ethical approval.

*Questionnaire development*

To create a basic item pool for the COVI-Prim questionnaire, we searched the literature for studies investigating the role of general practice during past pandemics. The search revealed a number of topics, some of which had been grouped to form topic areas in the literature. New topic areas were created for topics that did not fit into one of these. Based on the literature review, semi-structured telephone interviews were carried out with GPs. The results were recorded in keywords and evaluated in terms of content and topic. After identifying new topics in a first series of surveys (n = 9), no further new topics were found in a second (n = 5). It was therefore assumed that all relevant topics had been identified.

The literature and interviews revealed the following topic areas:


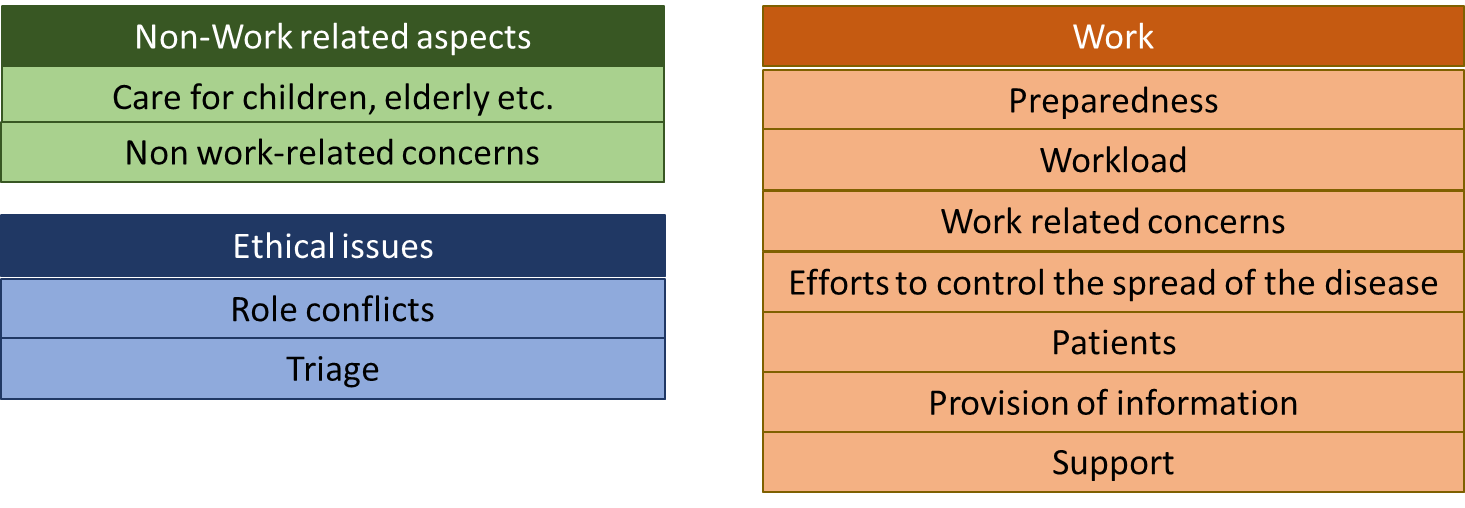


*S1 Figure. Topic areas according to literature review and interviews.*

Based on this structure, a questionnaire was developed that aimed to take all aspects into consideration, while being short enough to ensure a high response rate. The questionnaire was checked for comprehensibility by five GPs.

A short version of the questionnaire was prepared for a longitudinal survey. The items with potentially time-sensitive content were selected for the short version, as we assumed responses to these items might change during the pandemic. At the end of the full version of the questionnaire, each respondent had the opportunity to give his/her active consent to participate in the longitudinal survey (every 1 – 2 weeks) by providing their e-mail address.

*Structure of the Questionnaires*

The full questionnaire consisted of eight demographic items, 48 closed items (response scales: yes/no, yes/probably yes/probably no/no, very low/low/moderate/high/very high), three items requiring GPs to provide exact numbers (e.g. “How many COVID-19 tests did you perform last week?”), seven items requiring GPs to provide proportions (e.g. “How much of your overall working time was directly or indirectly linked to COVID-19?”), and five open-ended questions. The items in the questionnaire were grouped into seven sections: (1) demographic items, (2) preparedness at the beginning of the pandemic, (3) provision of information to GPs during the pandemic, (4) management of the pandemic by GPs, (5) personal worries, (6) personal emotions and (7) work content and burden of work. Overall, the questionnaire consisted of six pages.

To identify uncorrelated factors, exploratory factor analysis (VARIMAX rotation) was calculated for the following items: preparedness at the beginning of the pandemic, provision of information to GPs during the pandemic, management of the pandemic by GPs, and personal worries. To determine how many factors to retain, we applied Horn's parallel analysis and the criterion of eigenvalue > 1. Difficult items, defined as items for which more than 90% of responses fell into one of the two extreme categories, were excluded from the analysis. Ten factors had eigenvalues >1. Based on Horn’s parallel analysis, the original high number of factors fell to eight, with each explaining 3.0% to 15.2% of the variance (total variance explained = 46.4%). After eliminating all items with double loadings (items loading on two factors within a range of .1) and the highest factor loadings ≤ .3, a version of the questionnaire with 39 items remained. No item had to be excluded because of too many responses in an extreme response category. One item was excluded because of a mismatch between the item and factor content. Each of the remaining 38 items was assigned to one of the eight factors, with the factors ultimately including three to seven items. Internal consistency (Cronbach’s Alpha) of these eight factors ranged from α = .48 to α = .85.

*S2 Table. Internal consistency of the factors used to evaluate the pandemic*

|  | Cronbach’s alpha |
| --- | --- |
| Perception of risk | .851 |
| Provision of information to GPs | .810 |
| Preparedness for a pandemic | .726 |
| Self-confidence | .593 |
| Testing suspected cases | .557 |
| Decrease in number of patient contacts | .567 |
| Efforts to control the spread of the disease | .483 |
| Protection of staff | .484 |

Reflecting the items contained within them, the factors were named as follows: (1) Preparedness for a pandemic, (2) Testing suspected cases, (3) Protection of staff, (4) Provision of information to GPs, (5) Perception of risk, (6) Self-confidence, (7) Decrease in number of patient contacts, (8) Efforts to control the spread of the virus in the practice. To calculate factor scores (*f_x_*), the mean score of the items was calculated for each scale. The resulting score, which ranged from 1 to 4, was linearly transformed to 0-10 for a better interpretability (*f_xneu_ = (f_x_ - 1) * 3^-1^ * 10*). Calculation of factor scores was only performed when fewer than 50% of items were missing. To evaluate the effect of calculating factor scores with missing values, factor scores calculated form a complete response set analyzed. For this purpose, within the complete response set, single responses were randomly deleted (response set with missing values) from the original set of responses. The factor scores derived from the original response set (complete response set) was correlated with the factor score derived from the response set with missing values. The correlation between factor scores calculated with missing values and the factor score without missing values was r = .943 (95%CI: .938 -.947) for a seven-item scale with one missing item, r = .880 (95%CI: .869 - .889) with two missing items and r = .799 (95%CI .793 - .813) with three missing items.

The longitudinal questionnaire consisted of 15 closed items, three items required GPs to provide exact numbers, seven items required them to provide proportions, and five were open-ended items (3 pages). Of these 15 closed items, the factors “perception of risk” and “decrease in number of patient contacts” could be calculated. To ensure the response rate was high every week, the German version of the longitudinal survey was extended to include “hot topics”. The choice of hot topic was selected on the basis of weekly responses to the open-ended question “What was the biggest challenge you had to face as a GP this week?” A topic was then chosen, according to the previous week’s answers. The chosen topics were: Provision of information (survey period: 10.4. – 16.4 2020; response rate: 39%), Telemedicine (survey period: 17.4. – 23.4 2020; response rate: 55%), “Overlooked” patient groups (survey period: 24.4. – 30.4 2020; response rate: 45%), Residents of nursing homes for the elderly (survey period: 1.5. – 7.5 2020; response rate: 44%), Economic consequences for GPs (survey period: 8.5. – 21.5 2020; response rate: 39%), Personal evaluation of the lockdown (survey period: 22.5. – 4.6 2020; response rate: 38%), Lessons learned (survey period: 5.6. – 18.6 2020; response rate: 39%), and Evaluation of the role of GPs during the pandemic (survey period: 19.6. – 2.7 2020; response rate: 30%). Based on the comments of the GPs, an expert group created and discussed the items (GPs, Psychologist).

In Australia, follow-up surveys were performed on 23^rd^ May (response rate: 20 out of 21 contacted GPs), 6^th^ June (response rate: 13 out of 15), 20^th^ June (response rate: 7 out of 10), 4^th^ July (response rate: 5 out of 5), 18^th^ July (response rate: 1 out of 3) and 1^st^ August (response rate: 0 out of 1%).

*Translation*

The final baseline and longitudinal questionnaires were translated into English, Hungarian, Slovenian and Italian. The translation process for each language consisted of a translation (German to target language), followed by a back translation (target language to German). The back-translated version was compared to the original German version by a psychometrician, and the target language version was adapted where necessary.

*Survey*

The long version of the questionnaire and the short version of the questionnaire were transferred to LimeSurvey in all five languages. Since the developmental process of the translated questionnaires took time, the German version was the only one to be distributed at the beginning of April 2020 (COVI-Prim-*Start*; responses: Germany: 3^rd^ April – 27^th^ May, Austria: 3^rd^ April – 29^th^ May). The open survey began in the second half of April in Italy (23^rd^ April – 6^th^ May), at the beginning of May in Australia (8^th^ May – 8^th^ August), at the beginning of June in Slovenia (1^st^ June – 6^th^ July) and Hungary (5^th^ June – 2^nd^ July), and at the beginning of July in Switzerland (7^th^ July – 4^th^ August ) (Appendix Figure 2). Participation was voluntary and participants received no incentives.


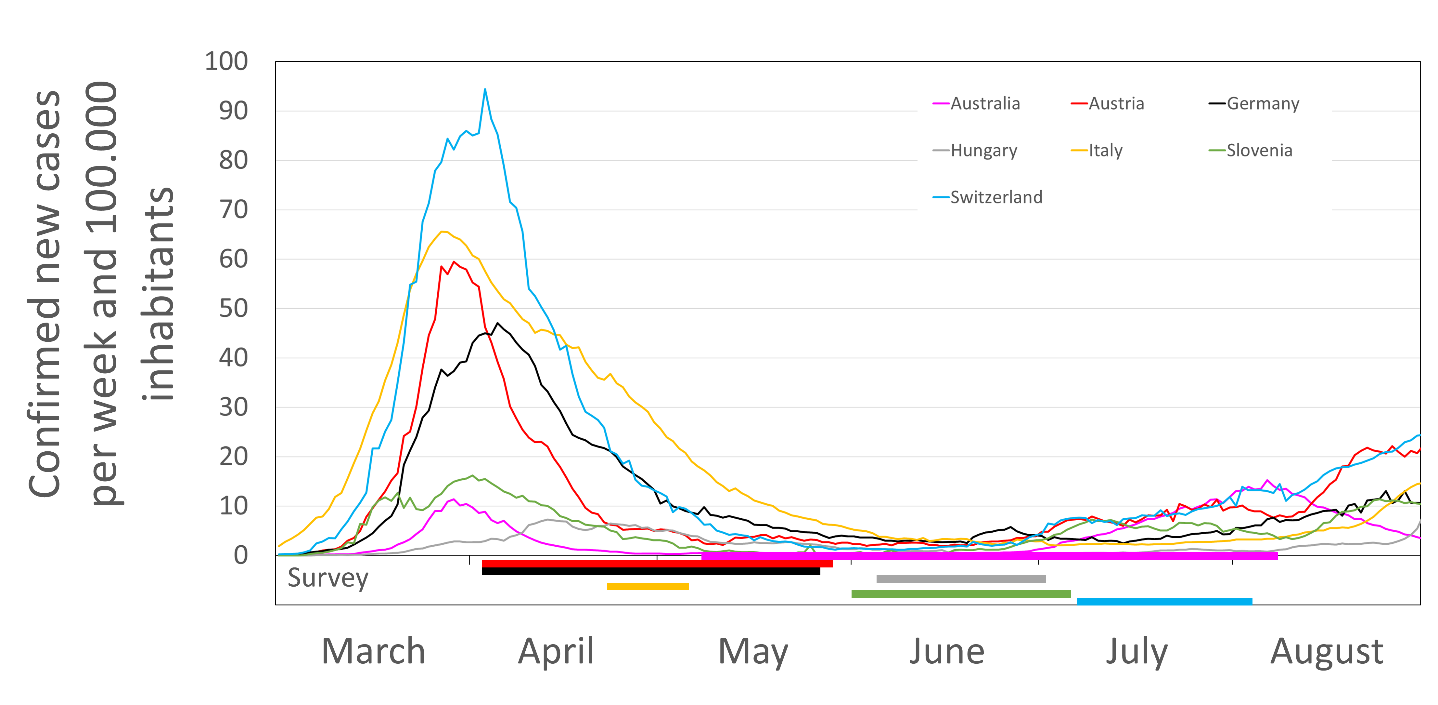


*S2 Figure. Confirmed new cases per week and 100,000 inhabitants from 1^st^ March until 31^st^ of July. timeline of survey in each country.*

*(Source:* [*https://covid19.who.int/table*](https://covid19.who.int/table) *; 1^st^ September 2020)*

Invitations to respond to the questionnaire were sent out by participating universities (Australia: Bond University, Queensland; Austria: Graz, Salzburg, Innsbruck; Germany: Frankfurt, Bochum, Hannover, Marburg, Gießen, Dresden, Freiburg, LMU Munich, Muenster, Aachen; Slovenia: Maribor; Switzerland: Bern) to GPs in their mailing lists. Local general practice associations (Styrian, Tyrolean, Carinthian, Vienna Society for General Practice), the Association of General Practitioners in Bavaria, Lower-Saxony and Baden-Wuerttenmberg and Austria. Michael Kochen of DEGAM-Benefits and the Austrian Forum for Primary Care (OEFOP) also invited their members to participate. In accordance with data protection regulations, the study team did not have direct access to the mailing lists. As these lists are likely to overlap, it is not possible to know the exact number of contacted GPs. A calculation of response rate is therefore not possible. Items were presented in a non-randomized manner. Some items for the “hot topics” were presented adaptively (e.g. different items were presented depending on whether a GP said she or he was the owner of the practice). No completeness check was performed before submission of the questionnaires. Participants could not review and change their responses after they had completed a page and started to respond on the next. Neither cookies, IP checks nor log file analyses were used to identify multiple entries. Atypical timestamps were not used to delete questionnaires responses. At the beginning of the survey, participants were informed about the length of the survey, who the investigator was, and the purpose of the study. Furthermore they were informed about the management of their data (which data, where and how long they are stored, access to the data). Before participants could start to answer the items, they had to state, that they have read this information and gave consent. After ending the survey, all data on the online platform were stored in SPSS files. GPs were offered no incentive or reward for their participation.

The median time required to answer the questionnaire was 11:00 minutes (interquartile range: 7:36 – 15:08) in Australia, 14.1 minutes (IQR: 10.5 – 20.2) in Austria, 13.4 minutes (IQR: 9.8 – 19.0) in Germany, 16.4 (IQR: 12.8 – 27.6) in Hungary, 17.3 (IQR: 12.0 – 22.5) in Italy, 11.2 minutes (IQR: 8.0 – 15.7) in Slovenia and 11.9 minutes (IQR: 9.0 – 18.3) in Switzerland. The completion rate of the survey ranged from 63.3% in Slovenia to 91.7% in Australia (Italy: 66.1%, Hungary: 67.3%, Austria: 79.7%, Germany: 85.2%, Switzerland: 87.8%).

*Ethics*

The study protocol was approved by the local ethics committee of Goethe University Frankfurt, Germany (ethics committee number 20-619), Bond University, Australia and Slovenia. According to national laws in Austria, Italy, Hungary and Switzerland no approval of the local ethics committee was necessary.

1. This section „Supporting information: Study design“ was already published in: Siebenhofer, A; Huter, S; Avian, A; Mergenthal, K; Schaffler-Schaden, D; Spary-Kainz, U; Bachler, H; Flamm, M COVI-Prim survey: Challenges for Austrian and German general practitioners during initial phase of COVID-19.

   PLoS One. 2021; 16(6): e0251736-e0251736. Doi: 10.1371/journal.pone.0251736 [↑](#footnote-ref-1)
